# Haplotype Analysis of Chloroplast Genomes for Jujube Breeding

**DOI:** 10.3389/fpls.2022.841767

**Published:** 2022-03-10

**Authors:** Guanglong Hu, Yang Wu, Chaojun Guo, Dongye Lu, Ningguang Dong, Bo Chen, Yanjie Qiao, Yuping Zhang, Qinghua Pan

**Affiliations:** Key Laboratory of Biology and Genetic Improvement of Horticultural Crops (North China), Ministry of Agriculture, Beijing Engineering Research Center for Deciduous Fruit Trees, Institute of Forestry and Pomology, Beijing Academy of Agriculture and Forestry Sciences, Beijing, China

**Keywords:** *Ziziphus jujuba*, chloroplast genome, genomic structure, phylogenetic analysis, evolutionary relationship, breeding strategy

## Abstract

Jujube (family Rhamnaceae) is an important economic fruit tree in China. In this study, we reported 26 chloroplast (cp) sequences of jujube using Illumina paired-end sequencing. The sequence length of cp genome was 161, 367–161, 849 bp, which was composed of a large single-copy region (89053–89437 bp) and a small single-copy region (19356–19362 bp) separated by a pair of reverse repeat regions (26478–26533 bp). Each cp genome encodes the same 130 genes, including 112 unique genes, being quite conserved in genome structure and gene sequence. A total of 118 single base substitutions (SNPs) and 130 InDels were detected in 65 jujube accessions. Phylogenetic and haplotype network construction methods were used to analyze the origin and evolution of jujube and its sour-tasting relatives. We detected 32 effective haplotypes, consisting of 20 unique jujube haplotypes and 9 unique sour–jujube haplotypes. Compared with sour–jujube, jujube showed greater haplotype diversity at the chloroplast DNA level. To cultivate crisp and sweet fruit varieties featuring strong resistance, by combining the characteristics of sour-jujube and cultivated jujube, three hybrid combinations were suggested for reciprocal crosses: “Dongzao” × “Jingzao39,” “Dongzao” × “Jingzao60,” “Dongzao” × “Jingzao28.” This study provides the basis for jujube species’ identification and breeding, and lays the foundation for future research.

## Introduction

Jujube (*Ziziphus jujuba* Mill.) is woody plant native to China, distributed throughout the country, that has been cultivated thousands of years there, and is now also grown across Asia, Europe, and the Americas (Guo et al., 2011). Jujube fruit is sweet and rich in vitamin C. Accordingly, Chinese jujube has important economic value as a food and a resource for the production of medicines and other health-promoting products (Shan et al., 2019; Rashwan et al., 2020; Sabri et al., 2021). Although there are extremely rich germplasm resources for jujube, the lack of improved varieties remains a limiting factor for its industrial development (Chang, 2018; Liu P. et al., 2020).

There are 700 jujube varieties and 30 sour-jujube varieties included in *Chinese fruit trees record–Chinese jujube* (Qu and Wang, 2013). Among them, 261 are suitable fresh-eating varieties, 224 are dried-fruit varieties, 159 are concurrent varieties and 56 candied date varieties. However, none of these varieties were obtained through cross breeding, having instead been selected from local varieties through long-term natural variation. So far, few novel varieties have been successfully bred through cross breeding. The breeding structure of jujube in China is characterized by disequilibrium. Among the existing production area and yield of jujube, about 80% of each is used for dried fruit (“dates”) and processing, with less than 20% of each used for fresh-fruit consumption. Special processing of certain varieties still lacks research and development. The degradation of local traditional jujube varieties is serious, leading to uneven quality and poor resistance against disease (Wang et al., 2012).

Both objective and subjective factors currently hinder the development of jujube hybrid breeding programs. *Objective factors*: First, jujube flowers are small, generally 5–7 mm in diameter, which makes it difficult for anthers and stamens to be manually peeled off; hence, both emasculation and artificial pollination are challenging. Second, the fruit-setting rate of jujube trees is very low, with a blooming period of 2 months in which flowers are abundant. The buds, flowers and fruits coexist during this blooming period. The nutritional competition among these parts is fierce, resulting in less effective flowers, a short effective pollination period, and serious flower and fruit drops. Accordingly, the natural fruit-setting rate of jujube is 1–2%, and likewise low for artificial hybridization. Third, jujube’s embryo abortion rate is high, and its kernel rate is low. Embryo abortion is prevalent in many varieties of jujube; this causes fruit drop, while late embryo abortion leads to no seed kernel formed, a crucial factor affecting artificial hybrid breeding. *Subjective factors*: Breeders tend to be bound by traditional concepts of hybrid breeding. At its core, artificial hybridization is the artificial selection of parents. Artificial emasculation, fertilization, and bagging are the breeding methods used for hybrid offspring, which ignores an in-depth understanding of the nature of hybrid breeding, impeding innovation, which seriously hinders the advancement of jujube hybrid breeding programs (Li and Wei, 2013; Wang et al., 2016).

Furthermore, systematic records during the breeding or vegetative propagation of jujube hybrids and/or clones are scarce. Additionally, for those breeding activities whose priority registration is that of new high-performing clones, it is absolutely essential have clear, unequivocal species identification.

There are two main ways to study biological evolution: (1) classical taxonomy based on apparent traits, and (2) biochemical and molecular analyses. Morphological markers are appearance traits that exhibit clear genetic polymorphisms (Li et al., 2015). Li et al. (2015) proposed an adaptive relationship exists between jujube leaf veins and temperature and precipitation, and argued that leaf vein characteristics could be used as a key feature to distinguish varieties and determine their patterns of introduction into different regions. However, quantitative traits are susceptible to external factors, and so they cannot fully convey the genetic variation harbored within a species. Molecular marker technology has since become the main method by which analyze genetic diversity, and now widely used in jujube genetic diversity research. In this respect, simple sequence repeat (SSR) markers have the advantages of stability, repeatability, and co-dominance. An example of their use is the work by Zhang et al. (2013), who used five pairs of SSR primers to analyze the genetic diversity and population structure of 50 different jujube samples collected along the Yellow River in Shaanxi Province.

For a long time, the taxonomic status of jujube and sour-jujube has been controversial (Li et al., 2018; Wang et al., 2019; Liu M. et al., 2020). To address these questions, many studies have been carried using molecular markers to analyze the nuclear genome, including those focusing on the haplotype composition, genetic diversity, and geographical origin of jujube (Liu et al., 2014; Shen et al., 2020; Song et al., 2021). Notably, chloroplast microsatellite markers were used to analyze cytoplasmic inheritance. For example, using chloroplast SSR technology (Yang, 2014) found that jujube and sour -jujube shared multiple haplotypes, with no significant genetic differentiation between these two populations, which suggests the evolution of sour-jujube into jujube occurred *via* multiple paths. Shen (2016) used nuclear and chloroplast SSR markers to comprehensively analyze polymorphism information, uncovering rich genetic diversity in Chinese jujube that was greater in sour-jujube than jujube at the nucleoplasm level. Yet, relatively few studies have conducted detailed genetic analyses of jujube vis-à-vis sour-jujube. Here, we present the complete and annotated DNA sequences for the chloroplast genomes of *Ziziphus jujuba* (jujube) and *Z. jujuba* var. *spinosa* (sour-jujube).

Chloroplast is one pigment body in algae and green plants, being a semiautonomous organelle in which photosynthesis occurs in plant cells. It has its own independent genome that encoded a series of specific proteins, and contains independent genetic materials and systems. Further, the chloroplast is also involved in the biosynthesis of fatty acids, vitamins, pigments, and amino acids, and thus critical for developmental processes that include these compounds (Prabhudas et al., 2016).

The chloroplast genome is a circular DNA molecule in angiosperms that has a typical tetragonal structure and consists of two inverted repeats (IR), a short single copy sequence (SSC), and a long single-copy sequence (LSC). Changes in the size of the chloroplast genome during evolution are mainly due to the extension, reduction, or loss of the IR regions and changes in the length of intergenic regions. The chloroplast genome is small in size, ranging from 120 to 217 kb. Compared with the nuclear and mitochondrial genomes, the chloroplast genome is more conserved in both its gene content and physical structure (Raubeson and Jansen, 2005). The nucleotide mutation rate of chloroplast genes is moderate, being higher than that of mitochondrial genes but lower than that of nuclear genes (Dong et al., 2020). Evolutionary events, such as gene mutation, duplication, loss, and rearrangement, have been detected in chloroplast genomes (Dong et al., 2013; Choi et al., 2016). The chloroplast genome is haploid, non-recombining, and maternally inherited, rendering it an ideal model for evolutionary and comparative genomic research (Birky, 2001; Wu and Ge, 2012). At a higher classification level, the comparative analysis of chloroplast genomes is proving useful for phylogenetic studies (Abdullah et al., 2020, 2021) and understanding genome evolution as related to changes in genome size, gene and intron loss, and nucleotide substitution (Ahmed et al., 2020; Lee et al., 2021). Comparative studies on chloroplast genomes have been conducted on multiple focus species (Young et al., 2011), genera (Greiner et al., 2008), and families of plants (Daniell et al., 2006).

Chloroplast genomes are reconstructed in phylogeny and DNA barcoding studies, and can be used to investigate the geographical origins of some important domesticated crops. However, there are relatively few studies that examine the genetic relationships of intraspecific varieties for breeding strategy (Daniell et al., 2016).

This study had four objectives: (1) To study the global structure of the chloroplast genome in jujube; (2) To analyze the polymorphism of nucleotide sequences and variation in repeat sequences among jujube chloroplast genomes; (3) To construct a haplotype network using these chloroplast genome sequences, to explore the phylogenetic relationships between jujube and sour-jujube; (4) To select parents for hybrid combinations of fresh-fruit jujube varieties. Our results will be useful for the identification of jujube cultivars, for determining their origins, for genetic breeding improvement and plant protection, and for conducting further evolutionary studies.

## Materials and Methods

### Plant Material Collection and DNA Extraction

We sequenced 26 jujube accessions in this study. All the sampled plants were cultured in the germplasm resources nursery of Institute of Forestry and Pomology, Beijing Academy of Agriculture and Forestry Sciences, Beijing, China. The plants were identified by Professor Qinghua Pan.

The specimens were stored in Key Laboratory of Biology and Genetic Improvement of Horticultural Crops (North China), Ministry of Agriculture. Each fresh leaf was immediately dried and stored with silica gel before its DNA extraction. Total DNA was extracted from 26 leaf samples using a modified CTAB protocol (Li J. et al., 2013). The high-quality total DNA samples were then stored at -80°C until their use.

Including the other 39 data downloaded from the Sequence Read Archive database (NCBI), a complete sample list can be found in Supplementary Table 1. All of 65 materials, corresponding to 52 cultivated jujube and 13 wild sour-jujube, were included in the analyses below.

### DNA Fragmentation, Library Preparation, and High-Throughput Sequencing

Total DNA was fragmented by ultrasonication, and 350 bp DNA fragments were recovered by gel cutting. Libraries with an average length of 350 bp were constructed using the Nextera XT DNA Library Preparation Kit (Illumina, San Diego, CA, United States). Sequencing was performed on the HiSeq X Ten PE150 platform (Illumina).

### Data Assembly

The raw data obtained from high-throughput sequencing were subjected to quality control procedures, by using Trimmomatic v0.36 (Bolger et al., 2014), after which SPAdes v3.10 was used for the *de novo* data assembly (Bankevich et al., 2012). The BLAST program was used to select chloroplast genome contigs from the assembled data (Altschul et al., 1997). Next, the chloroplast genome contigs were assembled using Sequencher 4.10.^1^ Published original second-generation sequencing raw data were downloaded from the Sequence Read Archive (SRA) database (refer to Supplementary Tables 1, 2) and chloroplast genome assembly was performed.

### Chloroplast Genome Annotation

Annotations was performed using the Plann script (Huang and Cronk, 2015), with the published chloroplast genome of *Z. jujuba* (GenBank number KX266829) serving as the reference sequence. Some unsuccessfully annotated or incorrectly annotated genes were manually added using Sequin software.

### Analysis of Variation Within Species

The chloroplast genome sequences of all jujube materials were assembled and aligned using MAFFT v7 software (Katoh and Standley, 2013). Informative sites and variable sites in the whole chloroplast genome, IRs, LSC, and SSC regions were counted by MEGA V7.0 for the comparison and alignment of sequence matrices (Sudhir et al., 2016). The nucleotide diversity, number of haplotypes, and haplotype diversity of the sequences were calculated using DnaSP V6 (Rozas et al., 2017). The orientation of single base substitutions (SNPs) and insertions/deletions (InDels) in each chloroplast genome is based on RBZ12. InDels were classified as described elsewhere (Dong et al., 2020).

### Phylogenetic Analysis

The chloroplast genomes of jujube and sour-jujube were compared to determine their phylogenetic relationships using the maximum likelihood method (ML). The ensuing ML trees were analyzed using RAxML v.8.2 software (Alexandros, 2014) with the evolution model set to “GTR-GAMMA.” A heuristic search was used to find the best tree, for which the support rate of each node was determined *via* 500 rounds of fast self-expansion analysis.

### Haplotype Network Construction

The haplotypes of the chloroplast genomes were calculated using DnaSP v6 software, and PopART software was used to build the TCS network diagram (Leigh and Bryant, 2015).

### Phenotyping

Phenotypic traits of the jujube breeding materials were tested here according to China national industry standard test guide LY/T2190-2013 (Li X. et al., 2013). Their fruit quality indexes were also tested according to the national standard by Pony Testing International Group.^2^

## Results

### Chloroplast Genome Size

**FIGURE 1:**
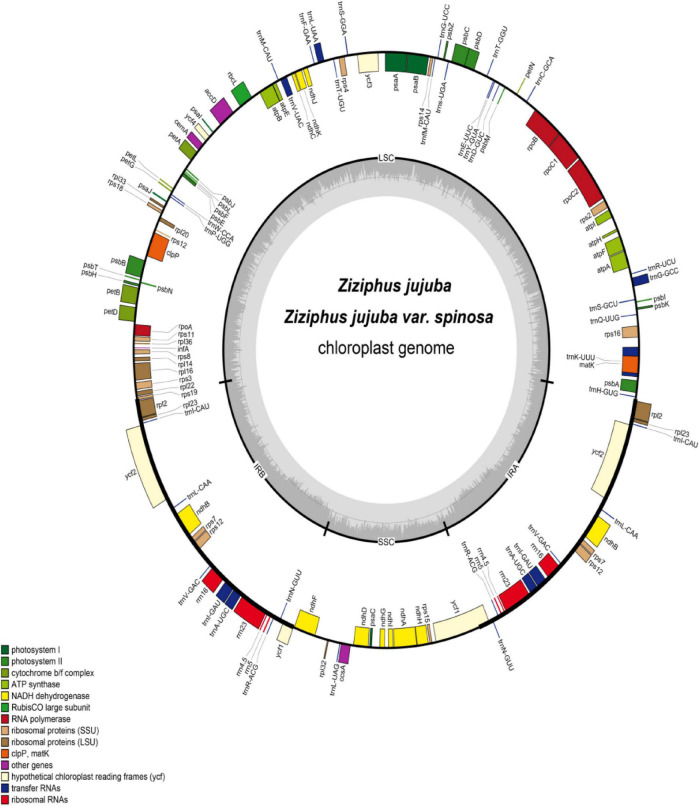
Map of the *Ziziphusjujuba* and *Ziziphusjujuba* var. *spinosa* chloroplast genomes. Genes marked inside the circle are transcribed clockwise; genes marked outside the circle are transcribed counterclockwise. Colored shading indicates the gene functional groups. Innermost dark gray corresponds to the GC content; and light gray corresponds to the AT content.

The chloroplast genome sizes of the 65 jujube materials analyzed in this study are shown in Supplementary Table 3. A genome’s total sequence length ranged from 161,367 to 161,849 bp. The size of the LSC region was 89,053 to 89,437 bp and that of the SSC region was 19,356 to 19,362 bp, while that of the IR (A/B) regions was 26,478 to 26,533 bp (Figure 1). Overall, the GC content of the chloroplast genomic sequences was consistent, at 36.7–36.8%, while that of the LSC, SSC, and IR (A/B) regions respectively were 34.5–34.6, 30.9, and 42.6% (Supplementary Table 3). Thus, the sequence lengths of the 65 chloroplast genomes were very similar, and their GC content almost the same. These chloroplast genome sequences have been added to the SRA under accession number (Supplementary Table 3).

A total of 130 genes were encoded in the chloroplast genome of jujube, including 112 unique genes. There were 18 genes in the IR region. Among the 112 unique genes, 76 were protein-coding genes, 4 were ribosomal RNA (rRNA) genes, and 30 were transfer RNA (tRNA) genes. Among the annotated genes, 17 contained introns, including 15 with a single intron (nine protein coding genes and six tRNA genes), and two having two introns each (*clpP* and *ycf3*). Among the 18 duplicated genes, there were seven protein-coding genes, seven tRNA genes, and four rRNA genes. The LSC region contained 60 protein-coding genes and 22 tRNA genes, while the SSC region had 12 protein-coding genes and one tRNA gene. Protein-coding genes in the chloroplast genome included nine encoding large ribosomal proteins (*rpl2, 14, 16, 20, 22, 23, 32, 33, 36*); 12 encoding small ribosomal proteins (*rps2, 3, 4, 7, 8, 11, 12, 14, 15, 16, 18, 19*); six encoding photosystem I components (*psaA, B, C, I, J, ycf4*), 15 encoding photosystem II components, and six encoding ATP synthase and electron transport chain components (*atpA, B, E, F, H, I*) (Table 1, Figure 1). The exons at the 5′ end of rps12 were positioned in the LSC region, and the repeat exons at the 3′ end were located in the IR region. Sequence analyses revealed that 45.66% of the chloroplast genome sequence encoded proteins, with another 1.73 and 5.60% that encoded tRNAs and rRNAs, respectively. The remaining 47.01% of the chloroplast genome consisted of introns, intergenic spacers, and pseudogenes.

**TABLE 1:** Genes in the jujube chloroplast genome.

Gene product	Gene product name	Gene name
1	Photosystem I	psaA, B, C, I, J, ycf4
2	Photosystem II	psbA, B, C, D, E, F, H, I, J, K, L, M, N, T, Z
3	Cytochrome b6/f	petA, B^a^, D^a^, G, L, N
4	ATP synthase	atpA, B, E, F^a^, H, I
5	Rubisco	rbcL
6	NADH oxidoreductase	ndhA^a^, B^a,^ ^c^, C, D, E, F, G, H, I, J, K
7	Ribosomal proteins (LSU)	rpl2^a,c,^ 14, 16^a^, 20, 22, 23^c^, 32, 33, 36
8	Ribosomal proteins (SSU)	rps2, 3, 4, 7^c^, 8, 11, 12^a, c, d^, 14, 15, 16^a^, 18, 19^c^
9	RNA polymerase	rpoA, rpoB, rpoC1^a^, rpoC2
10	Other proteins	accd, ccsa, cemA, clpP^b^, matK
11	Proteins of unknown function	ycf2^c^, 3^b^, ycf15^c^, infA^e^, ycf1^e^
12	Ribosomal RNAs	rrn4.5^c^, 5^c^, 16^c^, 23^c^
13	Transfer RNAs	trnH(GUG), K(UUU)^a^, Q(UUG), S(GCU), G(UCC)^a^, R(UCU), C(GCA), D(GUC), Y(GUA), E(UUC), T(GGU), S(UGA), G(GCC), fM(CAU), S(GGA), T(UGU), L(UAA)^a^, F(GAA), V(UAC)^a^, M(CAU), W(CCA), P(UGG), I(CAU)^c^, L(CAA)^c^, V(GAC)^c^, I(GAU)^a, c^, A(UGC)^a,c,^ R(ACG)^c^, N(GUU)^c^, L(UAG)

*^a^Gene containing a single intron;*

*^b^Gene containing two introns;*

*^c^Two gene copies in the irs;*

*^d^Gene divided into two independent transcription units;*

*^e^Pseudogene.*

### Chloroplast Genome Variability Analysis

Chloroplast genome variability was analyzed by detecting SNPs and InDels, which arise *via* translocation, inversion, and tandem duplication.

#### Number and Pattern of Single Base Substitution Mutations

We distinguished 118 SNPs: 85 in the LSC, 4 in the IRs, and 29 in the SSC (Supplementary Table 4). Of all the SNPs, 69 were in intergenic spacer regions, 37 in exon regions, and 12 in intron regions. ycf1 had the highest number of SNPs (7), followed by atpF (5). A total of 45 transitions (Ts) and 73 transversions (Tv) were detected, and their Tv-to-Ts ratio was 1:0.6, which indicating a bias toward transitions. The most frequently occurring SNP mutations were those of C to T and G to A; by contrast, mutations from C to G and from G to C were the least frequent.

#### Number and Pattern of InDel Mutations

**FIGURE 2:**
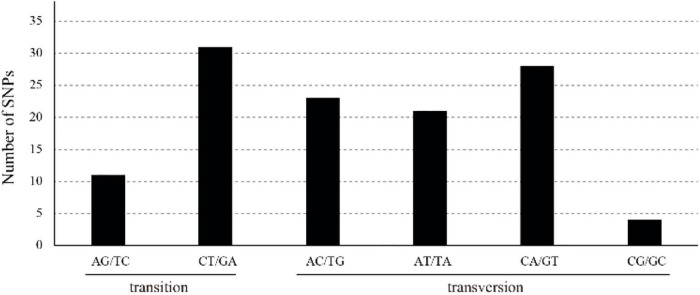
Pattern diagram of single base substitution (SNP) in *Z. jujuba* chloroplast genomes.

**FIGURE 3:**
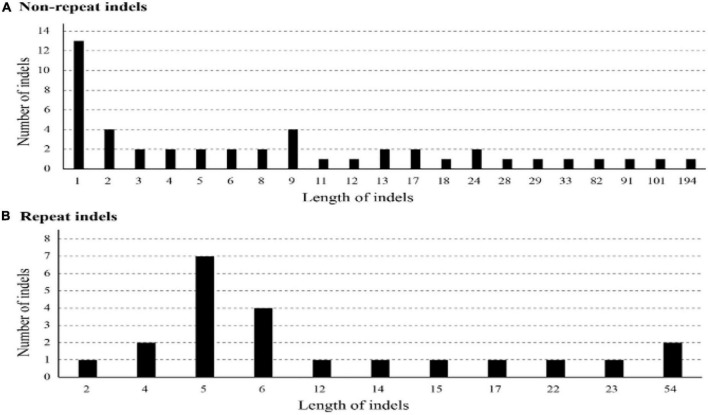
Number of non-repeat InDels **(A)** and repeat InDels **(B)** of each length in *Z. jujuba* chloroplast genome.

We detected 47 non-repeat InDels, two being in the IRs (*trnR, trnR-rrn5*) and 45 in the LSC (Supplementary Table 5). We found 36 gene spacers in one exon (*accD*) and eight introns (*atpF 1, ycf 32, trnL 5*). Most of these non-repeat InDels (85.1%) were 1–24 bp in length. We distinguished 22 repeat InDels, among which two were in the IRs (*ycf2-trnL, trnL-ycf2*) and 20 in the LSC. Nineteen gene spacers were identified in one exon (*trnL-ycf2*) and two introns (*ycf3*). Most of the repeat InDels (81.1%) were 1–17 bp in length. We found 61 SSR InDels: four in the IRs (*rps19-rpl2, rrn5-trnR, rpl2-rps19, trnR-rrn5*), 48 in the LSC, and nine in the SSC. Pattern diagram of SNP and distribution diagram of non-repeat indel and repeat indel were showed (Supplementary Table 5 and Figures 2, 3).

### Phylogenetic and Haplotype Network Construction

**FIGURE 4:**
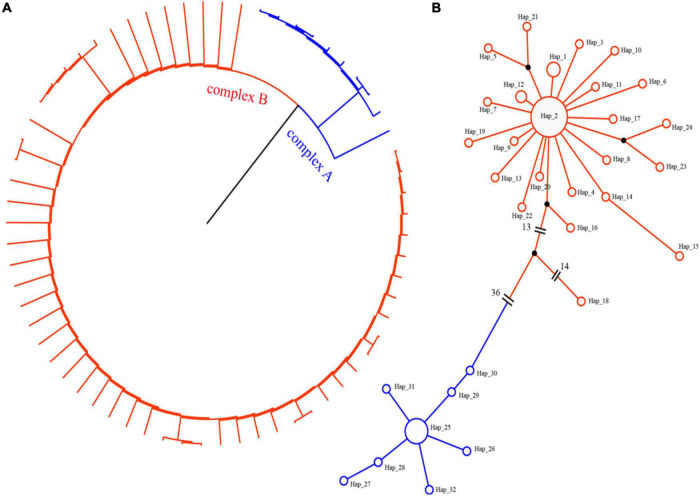
Haplotype network diagram and phylogenetic tree. **(A)** Phylogenetic tree. **(B)** Haplotype network diagram. Circles denote haplotypes. Circle size corresponds to the proportion of a specific haplotype out of 65 samples; red represents the jujube group, while blue represents sour-jujube group.

**FIGURE 5:**
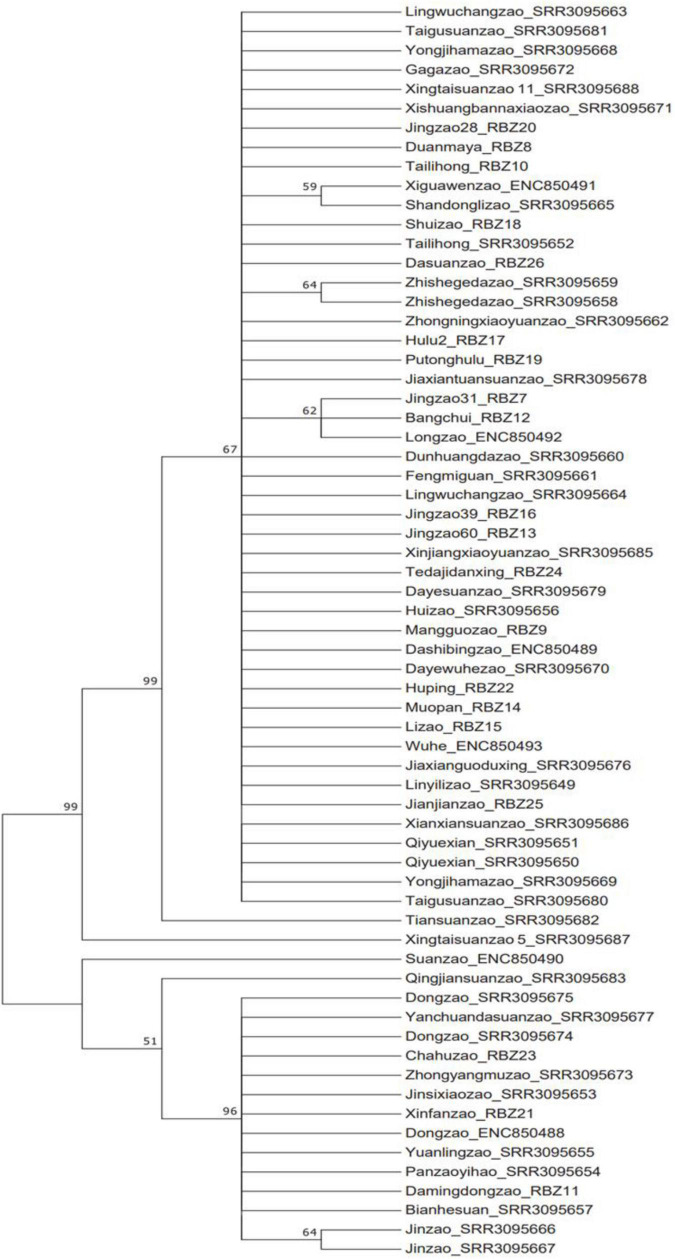
Phylogenetic tree of 65 jujube plastomes based on whole chloroplast genome sequences with maximum likelihood and Bayesian inference. Numbers near the nodes are values for bootstrap support.

Phylogenetic and haplotype network construction methods were used to analyze the origin and evolution of jujube and its sour-jujube. As Figures 4, 5 show, and as conveyed in Supplementary Table 6, we detected 32 effective haplotypes, consisting of 20 unique jujube haplotypes and 9 unique sour-jujube haplotypes.

## Discussion

### Chloroplast Genome Variability

Chloroplast genome variability was analyzed by detecting SNPs and InDels, which arise *via* translocation, inversion, and tandem duplication. The nucleotide mutation rate of chloroplast genes is moderate (Dong et al., 2020), and the evolutionary events have been detected in chloroplast genomes (Dong et al., 2013; Choi et al., 2016). Comparative studies on chloroplast genomes have been conducted on multiple focus species (Young et al., 2011), genera (Greiner et al., 2008), and families of plants (Abdullah et al., 2020, 2021; Ahmed et al., 2020; Lee et al., 2021), but, there are relatively few reports about chloroplast genome variability of intraspecific varieties (Daniell et al., 2016; Nock et al., 2019; Park et al., 2020).

In this study, We distinguished 118 SNPs, 47 non-repeat InDels, 22 repeat InDels and 61 SSR InDels among 65 jujube chloroplast genomes. Therefore, the genetic diversity of jujube chloroplast genome is higher compared with other species (Nock et al., 2019), suggesting that chloroplast genome sequences are indeed very well suited for the differentiation of jujube varieties.

### Evolutionary Relationship Between Jujube and Sour-Jujube

The chloroplast genome is haploid, non-recombining, and maternally inherited, rendering it an ideal model for evolutionary and comparative genomic research (Birky, 2001; Wu and Ge, 2012). In other research, such analyses have revealed copy number variations and rearrangements that were useful for phylogenetic reconstruction, DNA barcoding (Hollingsworth et al., 2011), and the investigation of geographical origins of some important crops (Delplancke et al., 2013).

Although individual gene segments on the chloroplast genome are already used for species differentiation in barcoding studies on plants, little is known about the usefulness of the entire chloroplast genome for intraspecies differentiation in general and for differentiation between modern varieties in particular. Results from the literature as well as from our own work suggest that chloroplast genome sequences are indeed very well suited for the differentiation of old varieties. On the other hand, they are less or not suitable for the genetic differentiation of modern cultivars, as they are often too closely related (Teske et al., 2020).

1. Because the chloroplast is highly conserved, the rate of mutation and generation of novel haplotypes occurs over thousands of years, resulting in a lower number of discoverable haplotypes, even over large sample areas (Cruzan and Hendrickson, 2020). In this study, We detected 32 effective haplotypes, resulting in a higher number of discoverable haplotypes, consisting of 20 unique jujube haplotypes and 9 unique sour-jujube haplotypes. Among them, 3 sour-jujube and 20 jujube were haplotype-2, one sour-jujube and one jujube were haplotype-19, 4 sour-jujube and 5 jujube were haplotype-25. Compared with sour-jujube, jujube displayed greater haplotype diversity at the chloroplast DNA level. Haplotype-18 (“Xingtaizao5”), 29 (“Qingjianzao”), and 30 (“Suanzao”) are transitional types between jujube and sour-jujube. In this study, the chloroplast genome was used to standardize the phylogenetic position of jujube germplasms (Figure 5). For the phylogenetic analysis of chloroplast genomes, ML and BI methods were implemented; both gave a near identical topological structure, with most nodes in either phylogenetic tree having high bootstrap support values.
2. Our chloroplast haplotype analysis revealed the germplasms of jujube can be clearly divided into two groups (Figure 4), sour-jujube (complex A) and jujube group (complex B). Although some varieties were named sour-jujube (suanzao, in Chinese), the maternal source was not sour-jujube and vice versa.
3. The study of human surnames is of great significance (Murci et al., 2001; Cláudia et al., 2021). Human surnames are basically determined by Y chromosome information. Especially, Chinese people have strict paternal surnames, which basically correspond to Y chromosome one by one (Chen et al., 2019; Zeng et al., 2019). Similarly, we used chloroplast genomic study to find the maternal surname for each jujube germplasm.

### Hybrid Breeding Strategy and Parents Selection Suggestion

Hybrid breeding is the most effective and conventional method used to breed fruit tree. Through sexual hybridization, separation and recombination of parental genetic material, and innovative genetic variation, hybrid breeding of fruit trees applies knowledge of plant genetic laws to cultivate new varieties with superior or comprehensive sought-after traits of parents. Further, it can use additive and non-additive genetic effects of the parents to maintain the genetic characteristics of hybrid types *via* asexual reproduction.

Advances in plant genome biology have inspired innovative approaches to expedite the progress of assembling desirable phenotypes in crop breeding programs. A set of haplotype-defining markers can provide crop breeders with an increased opportunity to attain optimized genetic combinations for improved plant performance (Bevan et al., 2017). In this respect, useful haplotypes were discovered for future breeding in rice (Abbai et al., 2019) and pigeonpea *Cajanus cajan* (Sinha et al., 2020). There is a need to track the inheritance patterns of haplotypes in crop breeding pedigrees. This is pivotal for assembling new genomic combinations because it helps to identify optimal parents for crosses that contain the desired combinations of traits or features (Varshney et al., 2021). Here, our analysis of chloroplast haplotypes from 65 jujube plastomes indicates that the varieties of jujube can be robustly separated into two groups. The haplotype map (Figure 4) provides us with a breeding navigation map which can help us make hybridization strategies according to the distance of kinship. Compared with selecting parents from single subpopulations, selecting parents from these two subpopulations can significantly broaden and augment the genetic diversity of the hybrid offspring.

The variety structure of jujube in China is still dominated by cultivars for making dry fruit products. Yet, cultivars bearing fruit for fresh consumption are gaining more and more recognition in the marketplace because of their rich nutritional components; not surprising, their planting coverage is also expanding rapidly. Fresh-consumption varieties of jujube are mainly late-maturing varieties, such as “Dongzao” and “Lizao,” and there is dearth of high-quality, medium and early maturing varieties. “Dongzao,” mainly distributed in the provinces of Shandong, Hebei and Shaanxi, is presently the largest fresh-consumption jujube variety in China. But this variety is beset by several limitations, under normal cultivation conditions, the northernmost line of cultivation is in Tianjin and Baoding (Hebei Province). The germination rate of “Dongzao” is low, and so is its fruit-setting rate is also low, though this can be increased by spraying the plants with gibberellin. The fruit-setting rate is low in the north, and the risk of frost is greater north of Beijing (Wang et al., 2012; Shi et al., 2020). Jingzao series varieties are bred by Institute of Forestry and Pomology, Beijing Academy of Agriculture and Forestry Sciences, and are deemed suitable for planting in northern China and western China, having the advantages of producing large-sized fruit, in high yields, at early maturity (Qinghua et al., 2011). According to the haplotype map (Figure 4) and mounting demand for jujube’s fresh fruits for eating and their breeding in northern China, we propose the following hybrid design.

**Hybrid design** (Phenotypic traits and the fruit quality indexes of breeding materials were obtained according to China’s national standard).

1. “Dongzao” × “Jingzao39”(reciprocal cross)
“Dongzao” Jujube features: The fruit is round, medium or large, normal fruit weight < 20 g, pulp is sweet, crisp, and good tasting; low natural fruit-setting rate and low fruit cracking rate; fruit matures in early and middle October. Each fruit has a total soluble sugar content of 22.2%, soluble solid content of 34.1%, titratable acid content of 0.43%, and vitamin C content of 352 mg/100 g.
“Jingzao39” features: Fruit is egg-shaped; normal fruit weight > 25 g, sour and sweet pulp, crisp, and good tasting, high natural fruit-setting rate, high fruit-cracking rate, fruit matures in mid and late September. Its total soluble sugar content is 21.7%, soluble solid content is 25.4%, titratable acid content is 0.36%, and the vitamin C content is 253 mg/100 g.
*Hope to obtain*: high natural fruit-setting rate, larger fruit than “Dongzao,” sour and sweet taste (i.e., sweeter than “Jingzao39”), low or non-cracking fruit rate, maturation at the end of September and in early October, and stronger resistance to diseases and insects.

2. “Dongzao” × “Jingzao60” (reciprocal cross)
“Jingzao60” features: Egg shape, fruit size > 25 g, slightly smaller than Beijing jujube 39. The pulp tastes sour and sweet, crisp, good taste, high natural fruit setting rate, fruit cracking rate is lower than “Jingzao39,” mature in mid and late September. The total soluble sugar content was 18.6%, the soluble solid content was 26.1%, the titratable acid content was 0.56%, and vitamin C content is 324 mg/100 g.
*Hope to obtain*: high natural fruit-setting rate, larger fruit than “Dongzao,” sour and sweet taste (i.e., sweeter than“Jingzao60”), low or non-cracking fruit rate, maturation in the end of September and in early October, and stronger resistance to disease and insect.

3. “Dongzao” × “Jingzao28”(reciprocal cross)
“Jingzao28” features: Fuit has an apple-like shape, with a size > 25 g; sour and sweet pulp, crisp, good tasting, high natural fruit-setting rate and high fruit-cracking rate, but both lower than “Jingzao39,” matures in mid and late September. Its total soluble sugar content is 21.6%, soluble solid content is 28.4%, titratable acid content is 0.41%, and vitamin C content is 275 mg/100 g.
*Hope to obtain*: high natural fruit-setting rate, larger fruit size than “Dongzao,” sour and sweet taste (i.e., sweeter than “Jingzao28”), low or non-cracking fruit rate, maturation at the end of September and in early October, and stronger resistance to disease and insect.

From a genomic point of view, the maternal lines of “Jingzao39,” “Jingzao60,”and “Jingzao28” were all derived from jujube group. Furthermore, “Jingzao39,” “Jingzao60,” and “Jingzao28” all had the same haplotype-2, which was the largest haplotype we identified. The “Dongzao” maternal line from sour-jujube group, has haplotype-25, this being the largest sour-jujube haplotype. Hybridization of sour-jujube and jujube is beneficial to inheriting the complementary advantages the parents offer for breeding commercial jujube.

## Conclusion

In this paper, we reported on 65 chloroplast genomes of jujube, including 26 chloroplast genomes sequenced in this study, and compared with other reported materials. The 65 jujube chloroplast genomes’ structure and gene content are similar, and highly conserved. In addition, variability of chloroplast genome (SNPs and InDels) was also analyzed. A haplotype network diagram was constructed; and a phylogenetic analysis was carried out.

Genetic relationships among jujube germplasms was revealed, which provides a timely basis for the selection of excellent cultivars. Three hybrid combinations were selected for reciprocal crosses: “Dongzao” × “Jingzao39,” “Dongzao” × “Jingzao60,” “Dongzao” × “Jingzao28.” The chloroplast genome results also verified the rationality of hybrid combinations. Overall, this study provides chloroplast genome sequences for further research on the identification and phylogeny of *Ziziphus jujuba*, and should help to enhance our overall understanding of the domestication history of *Z. jujuba* varieties.

## Data Availability Statement

The datasets presented in this study can be found in online repositories. The names of the repository/repositories and accession number(s) can be found in the article/Supplementary Material.

## Author Contributions

GH conducted the experiments, analyzed the data, and prepared the manuscript. YW analyzed the data. CG, DL, ND, BC, YQ, YZ, and QP performed the collection and processing of samples and analyzed the data. YZ and QP coordinated the experiments. All authors have read and agreed to the final version of the manuscript.

## Conflict of Interest

The authors declare that the research was conducted in the absence of any commercial or financial relationships that could be construed as a potential conflict of interest.

## Publisher’s Note

All claims expressed in this article are solely those of the authors and do not necessarily represent those of their affiliated organizations, or those of the publisher, the editors and the reviewers. Any product that may be evaluated in this article, or claim that may be made by its manufacturer, is not guaranteed or endorsed by the publisher.

## Footnotes

1
https://www.genecodes.com/

2
http://zx.ponytest.net.cn/
