# Corrigendum: Haplotype analysis of chloroplast genomes for jujube breeding

**DOI:** 10.3389/fpls.2023.1192452

**Published:** 2023-04-11

**Authors:** Guanglong Hu, Yang Wu, Chaojun Guo, Dongye Lu, Ningguang Dong, Bo Chen, Yanjie Qiao, Yuping Zhang, Qinghua Pan

**Affiliations:** Key Laboratory of Biology and Genetic Improvement of Horticultural Crops (North China), Ministry of Agriculture, Beijing Engineering Research Center for Deciduous Fruit Trees, Institute of Forestry and Pomology, Beijing Academy of Agriculture and Forestry Sciences, Beijing, China

**Keywords:** *Ziziphus jujuba*, chloroplast genome, genomic structure, phylogenetic analysis, evolutionary relationship, breeding strategy

In the published article, there was an error in the Funding statement. The correct grant numbers were missing from “the Presidential Foundation of Institute of Forestry and Pomology (Grant No. LGY202003)]”. The correct Funding statement appears below.

